# Projection of meteorological droughts in Nigeria during growing seasons under climate change scenarios

**DOI:** 10.1038/s41598-020-67146-8

**Published:** 2020-06-22

**Authors:** Mohammed Sanusi Shiru, Shamsuddin Shahid, Ashraf Dewan, Eun-Sung Chung, Noraliani Alias, Kamal Ahmed, Quazi K. Hassan

**Affiliations:** 1 Department of Water and Environmental Engineering, School of Civil Engineering, Faculty of Engineering, Universiti Teknologi Malaysia (UTM), 81310 Johor Bahru, Malaysia; 2 Department of Environmental Sciences, Faculty of Science, Federal University Dutse, P.M.B, 7156 Dutse, Nigeria; 3 School of Earth and Planetary Sciences (EPS), Spatial Sciences Discipline, Curtin University, Kent Street, Perth, Western Australia 6102 Australia; 4 Department of Civil Engineering, Seoul National University of Science and Technology, 01811 Seoul, Republic of Korea; 5 Department of Geomatics Engineering, University of Calgary, 2500 University Dr. NW, Calgary, AB T2N 1N4 Canada

**Keywords:** Climate sciences, Natural hazards

## Abstract

Like many other African countries, incidence of drought is increasing in Nigeria. In this work, spatiotemporal changes in droughts under different representative concentration pathway (RCP) scenarios were assessed; considering their greatest impacts on life and livelihoods in Nigeria, especially when droughts coincide with the growing seasons. Three entropy-based methods, namely symmetrical uncertainty, gain ratio, and entropy gain were used in a multi-criteria decision-making framework to select the best performing General Circulation Models (GCMs) for the projection of rainfall and temperature. Performance of four widely used bias correction methods was compared to identify a suitable method for correcting bias in GCM projections for the period 2010–2099. A machine learning technique was then used to generate a multi-model ensemble (MME) of the bias-corrected GCM projection for different RCP scenarios. The standardized precipitation evapotranspiration index (SPEI) was subsequently computed to estimate droughts from the MME mean of GCM projected rainfall and temperature to predict possible spatiotemporal changes in meteorological droughts. Finally, trends in the SPEI, temperature and rainfall, and return period of droughts for different growing seasons were estimated using a 50-year moving window, with a 10-year interval, to understand driving factors accountable for future changes in droughts. The analysis revealed that MRI-CGCM3, HadGEM2-ES, CSIRO-Mk3-6-0, and CESM1-CAM5 are the most appropriate GCMs for projecting rainfall and temperature, and the linear scaling (SCL) is the best method for correcting bias. The MME mean of bias-corrected GCM projections revealed an increase in rainfall in the south-south, southwest, and parts of the northwest whilst a decrease in the southeast, northeast, and parts of central Nigeria. In contrast, rise in temperature for entire country during most of the cropping seasons was projected. The results further indicated that increase in temperature would decrease the SPEI across Nigeria, which will make droughts more frequent in most of the country under all the RCPs. However, increase in drought frequency would be less for higher RCPs due to increase in rainfall.

## Introduction

Investigation of historical changes in climate variables has shown large variabilities over the past decades^1–4^. These variabilities have manifested in the frequencies, intensities, and risks of climatic hazards^5–8^, caused loss of lives^9^, destruction of properties^10^, devastation of ecosystem^11,12^, and damage to economies^13^ in many parts of the world. Studies have reported continuation or intensification in the changes of climate hazards in the future^14–17^. Therefore, it is imperative to understand future climate variations in order to develop appropriate mitigation and adaptation measures for the reduction of risk of damages and loss of lives and properties.

Drought is a highly devastating natural hazard that is projected to increase in many parts of the world due to higher variability of climate^18,19^. Droughts are classified as meteorological, agricultural, hydrological and social. But meteorological droughts aggravate all other kinds, and therefore, understanding it is crucial to develop mitigation measures. It occurs due to a reduction of precipitation or atmospheric water balance from long-term mean. As a result, it can occur in any climate regime due to natural variability of climate^20^. The increased variability in climate due to global warming has increased the frequency and severity of droughts in many parts of the globe in recent decades^21–25^, which are expected to swell with climate warming.

The African continent is considered as very prone to droughts due to high variability of rainfall. A large number of extreme droughts have been observed in the recent past which caused famines and loss of millions of lives^26^ in Africa. Studies reported more erratic behaviour of climate due to climate change, and thus, potentially increase its severity in different countries including Nigeria^9,27,28^. Numerous studies on the variability of climate and the changing nature of droughts using various indices have been conducted in Nigeria^3,29–39^. Though major drought events have not occurred in Nigeria for the past decades, studies showed an increasing tendency in climate variability, and thus, potential increase in drought frequency and affected areas^40–42^. The continuation of the present trend in droughts can have serious social, environmental, and economic consequences for the country. It would be highly devastating, if it continues to occur during growing seasons as economy and livelihood of majority of the population heavily rely on rain fed agriculture.

A large number of General Circulation models (GCMs) are publicly available in the Coupled Model Intercomparison Project Phase 5 (CMIP5)^43^ which have been widely used for drought projections. Even though CMIP5 GCMs are available at finer resolution compared to GCMs of CMIP3, they cannot directly be used at local level. Consequently, they need to be downscaled using statistical or dynamical methods. Statistical downscaling (SD) is widely used due to their advantage of cost and computational effectiveness, provision of point-scale climatic projections from the GCM-scale output, and the strength of incorporating observations directly into the methods^44–46^. However, using a large pool of GCMs for climate projection is unrealistic due to different degrees of uncertainties associated with different GCMs^47,48^. The selection of few suitable GCMs is therefore, suggested for generating multi-model ensemble (MME). As a result, mean projection for minimizing uncertainties is recommended^15,49^.

Realistic GCM selections for a region of interest can be conducted using various statistical indices and their combination^47,50,51^. Entropy has the ability of measuring average information content in one variable about another; therefore, it can be used for ranking of GCMs according to their ability to simulate annual and seasonal variability of observed climatic variables. This ranking information can be used in multi-criteria decision making (MCDM) framework for reliable GCMs selection, on the basis of the ability of GCMs to simulate historical precipitation and temperature, both spatially and temporally.

Though projection of climate over Nigeria has been assessed in a number of studies^40,52^, their impacts on droughts have not been taken into consideration, especially during different cropping seasons and using CMIP5 GCM ensemble. Different indices have been used in different regions of the world for the assessment of droughts. Among them, the standardized precipitation evapotranspiration index (SPEI)^53^ has been found highly effective, predominantly for semi-arid and arid environments because of inclusion of potential evapotranspiration (PET) in drought estimation, a factor of paramount importance for such regions^54^.

The major objective of this study was to project future changes in meteorological droughts during the growing seasons in Nigeria. For this purpose, GCMs were selected using a combination of entropy and MCDM methods. The selected GCMs were used for the projection of rainfall and temperature for three Representative Concentration Pathways (RCP), namely RCP 2.6, 4.5 and 8.5 in order to reveal changes due to different intensities of climate warming.

## Methods and materials

### Study area

**Figure 1:**
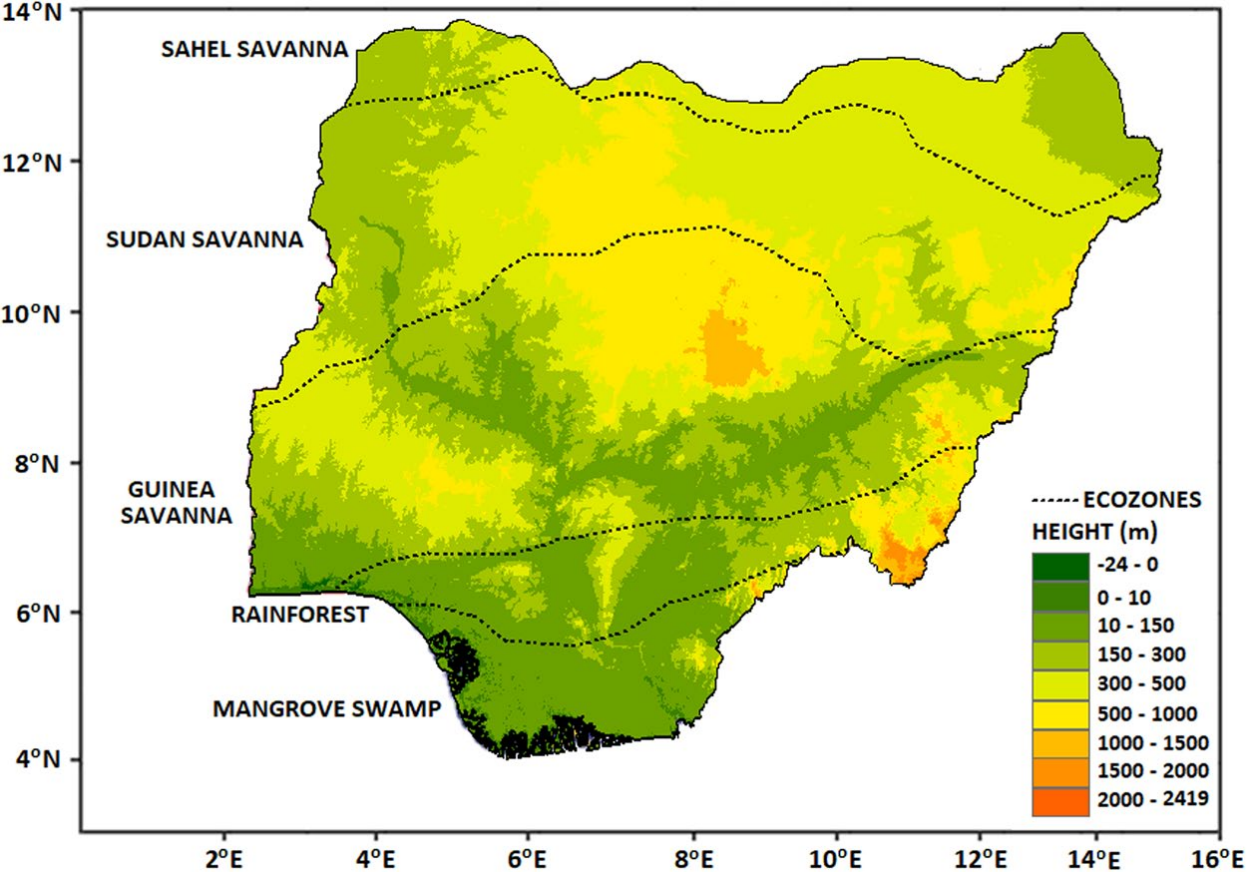
Topography and eco-climatic zones of Nigeria, showing the influence of annual precipitation.

Nigeria, (Latitude: 4^0^15′–13^0^55′N; Longitude: 2^0^40′–14^0^45′E) with an area of 923,000 km^2^ (Fig. 1), is located in the western part of Africa. The elevation of the country is mostly low, with the lowest elevation (0 m above mean sea level, msl) along the Atlantic coast in the south and the highest (2,419 m above msl) at Chappal Waddi in the northeast. The two main seasons are the dry and rainy seasons. There is a large variation in the climate from north to south. Most of the rainfall occurs between June and September in the semi-arid and arid north, and between April and October in the central and southern parts.

**Figure 2:**
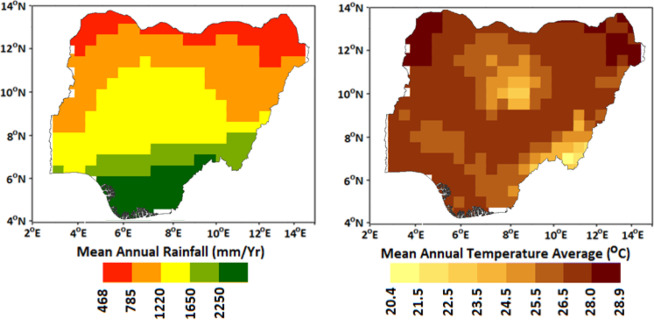
Spatial variation of mean annual Global Precipitation Climatology Centre (GPCC) rainfall (mm/y) and daily average Climatic Research Unit (CRU) temperature (°C) over Nigeria (1970–2000).

The annual average rainfall varies from more than 2,000 mm in the southern coast to less than 500 mm in the northern arid region (Fig. 2). On average, the temperature varies from 20.4 °C in the southeastern coastal region to more than 28 °C in the north (Fig. 2). The daily maximum temperature during the summer ranges from 30 °C to 37 °C in the south while it goes up to 45 °C in the north. On the other hand, daily minimum temperatures in wet season varies between 17 °C and 24 °C in the south but goes down to 12 °C in some parts of northern areas.

Nigeria is ecologically divided into five zones (from north to south) based on the amount of rainfall (Fig. 1). The Sahel Savanna is a warm desert climate, Sudan Savanna has a warm semi-arid. The tropical savanna climate and Guinea Savanna have a tropical savanna climate whilst rainforest has a mixture of tropical savanna and monsoon climate, and the Mangrove Swamp ecological zone experiences monsoon climate.

**Figure 3:**
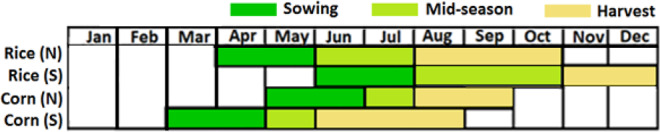
Growing periods of major crops in Nigeria.

The two major crops and their cropping seasons considered in this study are shown in Fig. 3. Spatially, the seasons for rice and corn vary from south to north. Therefore, the seasons for each crop are considered separately for each of the areas in this study. As the requirement of water is critical during sowing and mid-season, meteorological droughts during these two periods for each crop were assessed as well. For example, the SPEI in July was estimated for a time scale of four-month (April to July) to evaluate droughts during rice (N) growing season.

### Data

#### Gridded products

This study utilizes the Global Precipitation Climatology Centre (GPCC) monthly precipitation data of the Deutscher Wetterdienst^55^ and monthly average gridded temperature data from Climatic Research Unit (CRU)^56^ of the East Anglia University. Both of the products are at 0.5° × 0.5° resolution with a long temporal span. Rainfall and temperature data for a period of 1901–2015 were collected from 323 grid points to cover entire Nigeria. The data were used as reference for the selection of GCMs.

The GPCC precipitation product has a number of advantages: (1) wider period of data; (2) high data quality for climatological studies; (3) use of the highest number of precipitation records for product derivation; (4) time series completeness after January 1951^57,58^. The product is, therefore, frequently used in hydro-climatic investigations in many parts of the world, including Africa^59,60^.

The CRU, on the other hand, employs measurements from nearly 4,000 monitoring stations distributed across the globe. An extensive two stage manual and semi-automatic quality control measures are performed by the provider; the first is to ensure consistency, and the second involves removal of stations or months with large errors. Previous works show that the products are highly suitable for temperature studies in Africa^61,62^.

#### General Circulation Models (GCMs)

Temperature and rainfall of the CMIP5 GCMs were used in this study. The CMIP5, comprising historical and future climate simulations, is a globally coordinated GCM simulations dataset, obtained from different modeling groups^43^. Historical simulation of a total of 20 GCMs for the period 1961–2005 were obtained from the CMIP5 database. The availability of RCP2.6, 4.5 and 8.5 projections of both precipitation and temperature for Nigeria was considered for primarily selecting the GCMs. Major attributes of each of the GCMs used are presented in Table 1.

**Table 1:** Attributes of the general circulation models (GCMs) used in this study.

No	Institution	Model name	Resolution (Lon × Lat)
1	Beijing Climate Center, China Meteorological Administration	BCC-CSM1-1	2.8 × 2.8
2		BCC-CSM1.1(m)	1.125 × 1.125
3	National Center for Atmospheric Research, USA	CCSM4	1.25 × 0.95
4		CESM1-CAM5	1.25 ×0.95
5	Commonwealth Scientific and Industrial Research Organization, Australia	CSIRO-Mk3-6-0	1.875 × 1.875
6	The First Institute of Oceanography, SOA, China	FIO-ESM	2.8 ×2.8
7	Geophysical Fluid Dynamics Laboratory, USA	GFDL-CM3	2.5 × 2.0
8		GFDL-ESM2G	2.5 × 2.0
9		GFDL-ESM2M	2.5 × 2.0
10	NASA Goddard Institute for Space Studies	GISS-E2-H	2.5 × 2.0
11		GISS-E2-R	2.5 × 2.0
12	Met Office Hadley Centre, UK	HadGEM2-AO	1.875 × 1.25
13	Met Office Hadley Centre, UK	HadGEM2-ES	1.875 × 1.25
14	Institut Pierre-Simon Laplace, France	IPSL-CM5A-LR	3.75 × 1.875
15		IPSL-CM5A-MR	2.5 × 1.25
16	The University of Tokyo, National Institute for Environmental Studies, and Japan Agency for Marine-Earth Science and Technology	MIROC5	1.4 × 1.4
17		MIROC-ESM	2.8 × 2.8
18		MIROC-ESM-CHEM	2.8 × 2.8
19	Meteorological Research Institute	MRI-CGCM3	1.25 × 1.25
20	Norwegian Meteorological Institute, Norway	NorESM1-M	2.5 × 1.875

### Analytical techniques

#### Procedures

The procedures used in this study for the GCMs selection, downscaling of the GCMs simulations, preparation of MME and projection of drought are outlined below:

1. The 20 GCMs were re-gridded using bilinear interpolation to a common resolution of 2° × 2° and the CRU/GPCC data were aggregated to the same resolution for comparison;
2. The GCM simulations were compared with GPCC/CRU data using entropy-based methods to select a subset of 20 GCMs, based on their ability to replicate historical rainfall/temperature;
3. The simulations of the selected GCMs were re-gridded to a resolution of 0.5° × 0.5° using the bilinear interpolator and the bias in GCM simulated rainfall and temperature were corrected based on the GPCC rainfall and CRU temperature, respectively. Four bias correction methods, namely power transformation (PT), linear scaling (SCL), general quantile mapping (GEQM) and gamma quantile mapping (GAQM) were used to correct for systematic bias, inherent in the products;
4. The bias correction method was selected based on a set of statistical indices and the coefficients of the bias correction methods were used to correct the bias in the GCM simulations for RCP 2.6, 4.5 and 8.5 for the period of 2010–2100;
5. Random Forest (RF) was used for the generation of the MME of downscaled GCM projections;
6. The SPEI was estimated from the MME precipitation/temperature for all the three RCPs at each grid point (323 grid points), covering Nigeria, for different 50-year period with a time interval of 10-year during 2010–2100;
7. The SPEI for each growing season was fitted with the best probability distribution function (PDF) for the estimation of the return periods for different drought categories and different growing seasons;
8. Trends in projected rainfall, temperature, and SPEI were estimated using modified Mann-Kendall test (MMK) and their associations were examined using Kendall-tau correlation coefficient for different seasons for different 50 year periods.

#### GCM Selection using entropy-based methods and downscaling

Three entropy-based methods, namely symmetrical uncertainty (SU), gain ratio (GR), and entropy gain (EG), were used to assess similarity of GCM historical simulated rainfall with GPCC rainfall and temperature with CRU temperature for the period 1961 to 2005. The EG is an information-based concept, which is a measure of uncertainty of a random variable. It can be used for the estimation of information of one variable compared to another^63^. This capability of entropy is used for assessing the ability of GCM to simulate observed climate. The GR and SU are the modified versions of EG, which can overcome the bias in GR estimation to higher values. Details about the entropy methods used in this study can be found in Khan *et al*.^64^, Salman *et al*.^15^, and Shiru *et al*.^65^.

The MCDA was used for ranking of the GCMs according to EG, GR and SU values, estimated at different grid points over Nigeria. Higher weight was given to the GCMs that was found to attain a particular rank at most of the 323 grid points. For example, if a GCM was ranked top by an entropy measure at most of the grid point, it was given the highest weight and vice versa. The GCMs were ranked separately for EG, GR and SU and then averaged to get the final rank of the GCMs.

Four bias correction methods namely PT, SCL, GEQM and GAQM were used in this work for downscaling of selected GCM simulations. Biases of the selected models were corrected by comparing the GCM simulated rainfall/temperature with the GPCC rainfall/CRU temperature for historical period. Bias correction parameters were derived by comparing data for the period 1961–1992, and the parameters were then used to correct bias of the GCM simulations for 1993–2005. This was done to assess performance of the bias correction methods. Standard statistical indices namely, percentage of bias (Pbias), normalized root mean square error (NRMSE), Nash-Sutcliff efficiency (NSE), modified index of agreement (MD) and relative standard deviation (RSD) were considered. The best performing method was then selected for downscaling future rainfall and temperature of Nigeria.

#### Random forest for ensemble aggregation

The regression-based MME has the ability to preserve variance in the average of the MME, and is widely utilized in recent times. In this study, RF regression was used for the estimation of GCM ensemble. In RF, nonlinear relationship between the observed rainfall/temperature and the GCM simulated rainfall/temperature was generated. The outcome was then used for the preparation of ensemble of GCM projections. The RF is an effective and robust algorithm for generating the MME because: (1) it can avoid over-fitting; (2) many different types of input variables can be implemented without deleting and regularizing variable; and (3) it has analytical and operational flexibility.

#### Standardized precipitation evapotranspiration index (SPEI)

In computing the SPEI, water surplus or deficit for different time scales (D) was estimated from the difference of precipitation and PET^53^. The SPEI values were then estimated from the best-fitted distribution parameters of *D*. The values, ranging from −1.0 to −1.5, are considered moderate droughts, −1.5 to −2.0 are severe, while values > −2.0 are defined as extreme droughts^51^.

The calculation of PET for the estimation of the SPEI can be conducted using different methods including temperature, radiation, and mass transfer methods^66^. The SPEI is found more sensitive to radiation-based PET estimation and less to temperature-based proxy methods^67^. Begueria *et al*.^68^ observed that the use of different PET methods in semi-arid and arid regions results in significant differences in the SPEI series. They recommended Penman-Monteith method to be chosen for PET estimation, followed by the Hargreaves and the Thornthwaite methods. The Thorntwaite method, in comparison with other techniques, requires less number of meteorological variables for the estimation of PET, which makes it more suitable for PET calculations in data sparse locations such as Nigeria. For the determination of droughts of a season, last month SPEI value of the season was used.

The return periods (RP) of seasonal droughts were estimated from the SPEI values of different growing seasons. Droughts were defined if SPEI values were lower than −1.0. Hence, zero values were assigned to years with no drought. Droughts frequency analyses were conducted only on the non-zero values and corrections were made using non-exceedance probability (*F*′) in order to account for the zero values.

1 $$F{\prime} =\,q+(1-q)F$$

where, *F* is the non-exceedance probability value derived by the use of frequency analysis on the non-zero values, and *q* is estimable as the ratio of the number of years without drought events to the total number of years^25,69–71^.

#### Sen’s slope estimator

The rate of change in the SPEI, temperature, and precipitation was assessed using Sen’s slope estimator^72^, where change (*Q*_med_) is computed as the median of *N* slopes estimated from the consecutive two points of the series as follows:

2 $${Q}_{med}=\{\begin{array}{c}{Q}_{|\frac{N+1}{2}|}\,if\,N\,is\,odd\\ \frac{{Q}_{|\frac{N}{2}|}+{Q}_{|\frac{N+2}{2}|}}{2}\,if\,N\,is\,even\end{array}$$

#### Modified Mann-Kendall (MMK) test

Trends significance in precipitation, temperature, and the SPEI changes was carried out using the MMK test. This test was used considering its ability to separate natural variability of climate from unidirectional climate change due to global warming^57,73^. The classical Mann-Kendall test statistic (*S*) for a time series, *x* with *n* number of data points is calculated as^74^:

3 $$S=\mathop{\sum }\limits_{k=1}^{n-1}.\mathop{\sum }\limits_{i=k+1}^{n}{\rm{sign}}\,({x}_{{\rm{i}}}-{x}_{{\rm{k}}})$$

where,

4 $$sign\,({x}_{{\rm{i}}}-{x}_{{\rm{k}}})=\{\begin{array}{c}+1\,when\,({x}_{{\rm{i}}}-{x}_{{\rm{k}}}) > 0\\ 0\,when\,({x}_{{\rm{i}}}-{x}_{{\rm{k}}})=0\\ -1\,when\,({x}_{{\rm{i}}}-{x}_{{\rm{k}}}) < 0\end{array}$$

The significance of the trend is calculated using *Z* statistics as:

5 $$Z=\{\begin{array}{c}\frac{S-1}{\sqrt{Var\,(S)}}\,when\,S > 0\\ 0\,when\,S=0\\ \frac{S-1}{\sqrt{Var\,(S)}}\,when\,S < 0\end{array}$$

where, ${\boldsymbol{Var}}\,({\boldsymbol{S}})$ is the variance of *S*.

The significance of trend estimated by the use of MK test is first removed from the time series in MMK test^75^, then equivalent normal variants of rank (*R*_*i*_) of the de-trended series is estimated as:

6 $${Z}_{i}={\phi }^{-1}\,\left(\frac{{R}_{i}}{n+1}\right)$$

where, ${\phi }^{-1}$ is the inverse standard normal distribution function. The self-similarity correlation matrix of the time series or the Hurst coefficient (*H*) can be derived using the equation that follows^76^:

7 $${C}_{n}(H)=[{\rho }_{|j-i|}],\,for\,i=1:n;\,j=1:n$$

8 $${\rho }_{l}=\frac{1}{2}\,({|l+1|}^{2H}-2{|l|}^{2H}+{|l-1|}^{2H})$$

where, ${\rho }_{l}$ is the autocorrelation function of lag *l* for a given *H*. The value of *H* is obtained by the use of maximum log likelihood function. The significance level of *H* is determined by the use of mean and standard deviation for *H* = 0.5. If *H* is found significant, the biased estimate of the variance of *S* is calculated for a given *H* as:

9 $$V{(S)}^{H{\prime} }=\sum _{i < j}.\sum _{k < l}\frac{2}{\pi }{\sin }^{-1}\left(\frac{\rho |j-i|-\rho |i-l|-\rho |j-k|+\rho |i-k|}{\sqrt{(2-2\rho |i-j|)(2-2\rho |k-l|)}}\right)$$

The bias in estimation of $V{(S)}^{H}$ is removed using a bias correction factor, *B* as below:

10 $$V{(S)}^{H}=V{(S)}^{H{\prime} }\times B$$

where, *B* is a function of *H*. The significance of MMK test is computed using Eq. (5) by replacing $V(S)$ with $V{(S)}^{H}$.

## Results

### Selection of GCMs

The different weights of the GCMs obtained using EG, GR and SU according to their ability to replicate historical rainfall and temperature are given in Table 2. Average of the weights, obtained by different GCMs for rainfall and the temperature, were used to rank the GCMs (last column of Table 2). The MRI-CGCM3 was found to be the most capable of simulating climate of Nigeria followed by HadGEM2-ES, CSIRO-Mk3.6.0 and CESM1-CAM5. Therefore, these four GCMs were used to evaluate changing behavior of droughts over Nigeria associated with climate change.

**Table 2:** Calculation of average weights of each of the GCMs according to their capability to replicate historical rainfall and temperature (boldface rows indicates selected GCMs).

GCM	EG				GR				SU				Average WT			RK
	RN	Temperature			RN	Temperature			RN	Temperature			EG	GR	SU	
		Max	Avg	Min		Max	Avg	Min		Max	Avg	Min				
BCC-CSM1.1(m)	0.00	0.57	1.11	0.62	2.08	0.71	0.75	0.41	0.06	0.69	1.05	0.52	0.57	0.99	0.58	13
BCC-CSM1-1	0.00	0.37	0.85	0.05	0.00	2.03	0.87	0.25	0.00	0.64	1.00	0.14	0.31	0.79	0.44	18
CCSM4	0.02	1.36	0.61	0.00	1.32	0.99	0.19	0.08	0.46	1.09	0.54	0.00	0.49	0.65	0.52	15
**CESM1-CAM5**	2.41	4.70	6.41	3.96	0.86	4.03	5.26	1.18	1.37	4.71	6.28	2.82	4.37	2.83	3.80	4
**CSIRO-Mk3.6.0**	0.27	2.83	5.55	9.07	1.72	4.64	2.79	5.11	1.18	3.83	4.35	8.53	4.43	3.56	4.47	3
FIO-ESM	0.08	5.49	0.69	0.22	0.89	4.12	2.05	0.04	0.37	5.51	0.86	0.00	1.62	1.77	1.68	10
GFDL-CM3	0.81	0.39	1.37	2.43	4.87	0.84	2.47	2.14	3.03	0.51	2.00	2.88	1.25	2.58	2.10	6
GFDL-ESM2G	0.02	0.21	0.34	0.27	1.30	1.04	1.30	0.65	0.08	0.46	0.69	0.59	0.21	1.07	0.46	17
GFDL-ESM2M	0.54	0.46	0.54	0.74	2.06	0.63	2.21	1.02	1.32	0.40	1.09	0.74	0.57	1.48	0.89	12
GISS-E2-H	1.14	0.32	0.02	0.00	1.44	0.81	0.12	0.23	0.78	0.33	0.10	0.00	0.37	0.65	0.30	19
GISS-E2-R	0.87	0.58	0.11	0.00	1.18	1.20	0.13	0.16	1.07	0.78	0.09	0.00	0.39	0.67	0.48	16
HadGEM2-AO	1.97	2.68	4.93	0.36	1.60	1.22	3.39	1.10	2.36	1.51	4.96	0.31	2.49	1.83	2.28	5
**HadGEM2-ES**	5.21	6.18	6.96	7.06	1.90	2.03	4.68	4.74	3.53	4.22	6.46	7.88	6.35	3.34	5.52	2
IPSL-CM5A-LR	7.91	2.84	0.00	0.00	1.40	3.09	0.41	0.02	3.62	3.23	0.083	0.02	2.68	1.23	1.74	9
IPSL-CM5A-MR	4.81	1.40	0.72	0.00	3.21	1.83	1.14	0.08	5.34	1.63	1.06	0.00	1.73	1.56	2.01	7
MIROC5	0.06	1.22	1.83	1.92	0.00	1.43	1.95	2.81	0.00	1.18	1.84	3.89	1.26	1.55	1.73	8
MIROC-ESM	0.00	0.61	0.88	4.08	1.15	1.25	2.11	1.29	0.14	0.97	1.37	2.09	1.39	1.45	1.14	11
MIROC-ESM-CHEM	0.00	0.00	0.03	2.58	0.77	0.51	0.73	1.33	0.04	0.06	0.22	1.88	0.65	0.84	0.55	14
**MRI-CGCM3**	11.17	5.00	4.32	3.87	7.13	4.80	3.95	4.16	12.30	5.45	4.40	4.91	6.09	5.01	6.76	1
NorESM1-M	0.00	0.00	0.00	0.00	2.38	0.00	0.75	0.20	0.23	0.00	0.06	0.02	0.00	0.83	0.08	20

RN: Rainfall WT: Weight RK: Rank.

### Downscaling and projection of climate

The rainfall and temperature of the selected GCMs were interpolated at GPCC/CRU resolution and then the bias in GCM simulations were corrected using four bias correction methods, noted earlier. Five performance metrics were used for the assessment of performance of different bias correction methods^65^ during validation period (1993–2005). The results for downscaling rainfall, are presented in Table 3. This includes performance of raw GCM in simulating GPCC rainfall. It can be seen that (Table 3) SCL outperformed other metrics in downscaling rainfall and temperature for all the GCMs, except for some metrics of GAQM method for CSIRO-Mk3.6.0. Therefore, the SCL was chosen for downscaling of rainfall and temperature of selected GCMs and generation of the MME mean projection at each of the 323 GPCC/CRU grid points.

**Table 3:** Performance metrics of the selected bias correction methods for downscaling of rainfall.

GCM	Method	NRMSE	PBIAS	NSE	RSD	MD
CESM1-CAM5	GCM	80.5	−19.3	0.35	1.11	0.67
	SCL	**40.6**	**−2.5**	**0.83**	**0.97**	**0.83**
	GEQM	53.6	−100	−0.01	0	0.66
	PT	53.6	−2.9	0.71	0.92	0.75
	GAQM	46.7	−2.9	0.78	0.94	0.81
CSIRO-Mk3.6.0	GCM	80.7	−41.9	0.34	0.65	0.65
	SCL	45	**1.3**	0.80	0.93	0.81
	GEQM	100.5	−100	0	−0.02	0.65
	PT	48.6	1.9	0.76	0.97	0.78
	GAQM	**40.6**	2.3	**0.83**	**0.99**	**0.84**
HadGEM2-ES	GCM	40.9	−12.9	0.83	**0.98**	0.83
	SCL	**33.9**	**−7.0**	**0.88**	0.92	**0.86**
	GEQM	100	−100	−0.01	0	0.66
	PT	50.1	−8.8	0.75	0.85	0.78
	GAQM	41.4	−7.9	0.83	0.87	0.83
MRI-CGCM3	GCM	56.6	23.2	0.68	1.21	0.81
	SCL	**29.7**	**−0.3**	**0.91**	**1.01**	**0.88**
	GEQM	100	−100	−0.01	0	0.66
	PT	45.8	−1.6	0.79	0.96	0.79
	GAQM	38.1	2.4	0.85	0.94	0.85

The best statistics are boldfaced.

**Figure 4:**
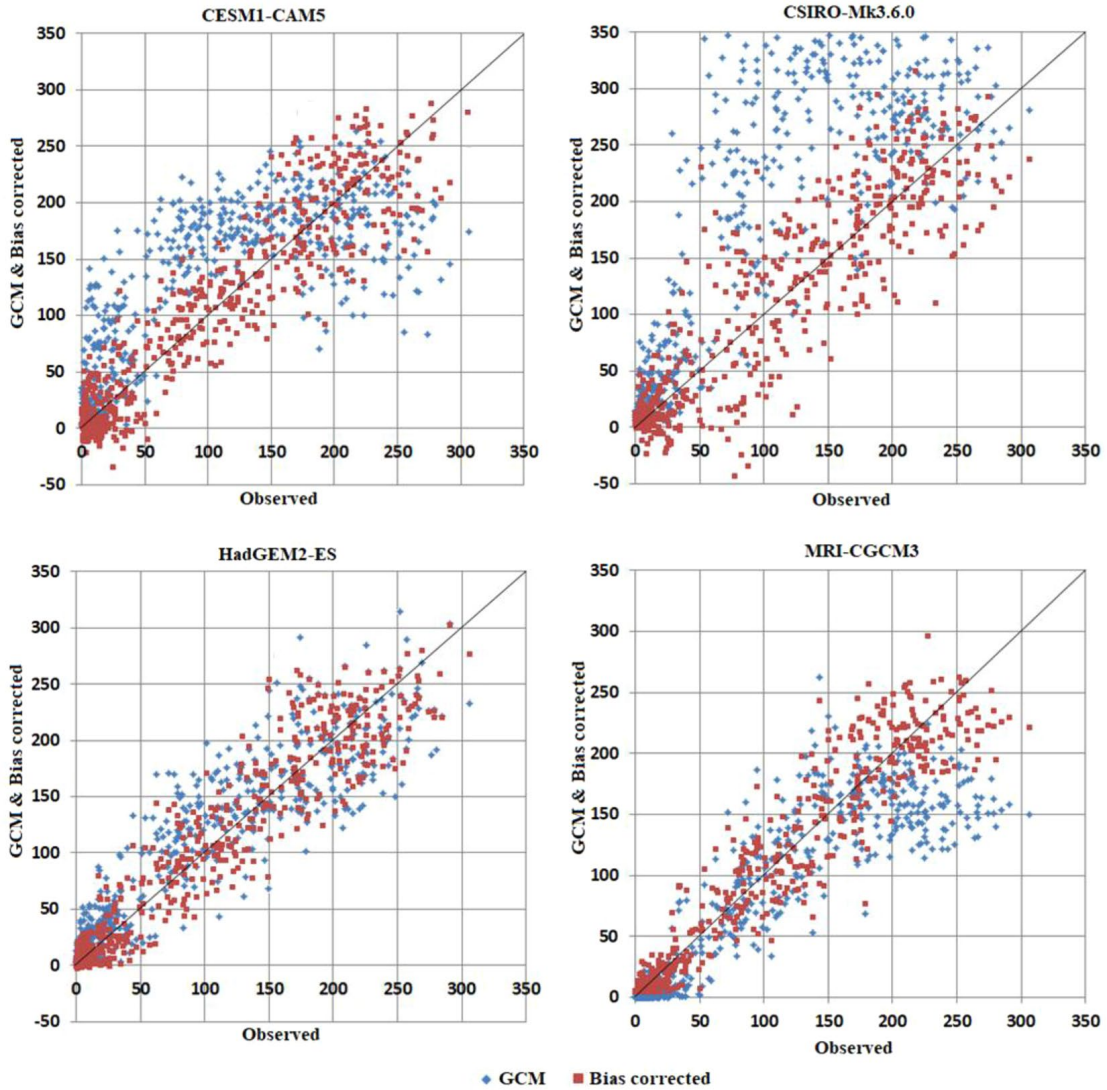
Scatter plots of mean monthly rainfall of selected raw GCMs and their bias corrections over Nigeria during 1961–2005.

To assess efficiency of the SCL method in correcting the bias of the GCMs, scatter plot was used (Fig. 4), which shows a stronger relation between observed and bias corrected rainfall, compared to simulated rainfall of the four selected GCMs. Similar relationship was noticed between observed and bias corrected temperature.

**Figure 5:**
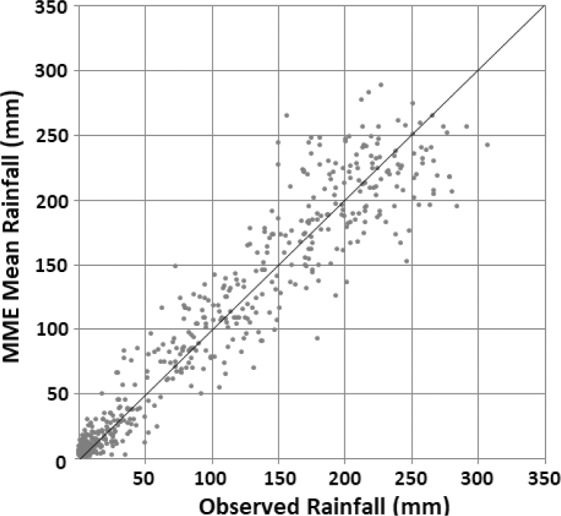
Scatter plot of RF estimated MME mean monthly rainfall and GPCC mean monthly rainfall, averaged over Nigeria for the period 1971–2005.

The scatter plots of the MME mean rainfall generated using the RF and that of the GPCC, averaged for the 323 grid points over Nigeria, for the period 1971–2005 is presented in Fig. 5. This shows a close relationship between the estimated MME and the GPCC mean rainfall. It further indicates a good performance of RF in MME mean rainfall computation. The correlation coefficient between the GPCC and the MME mean rainfall was in the range of 0.94 to 0.99 for all the grid points. It can, therefore, be concluded that estimated MME using the RF can improve the accuracy in the projection due to the fact that associated uncertainties with the individual GCMs can be reduced. The MME mean rainfall and the temperature were used for the estimation of future changes in droughts during different crop growing seasons of Nigeria.

### Projected trends in climate and drought index

**Figure 6:**
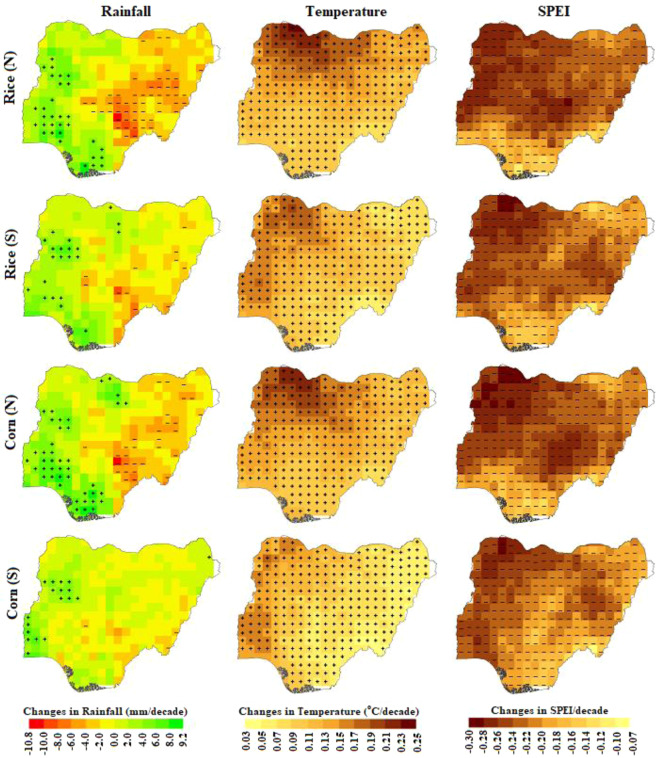
Spatial distribution of the trends in rainfall, temperature and SPEI for the period 1910–2099 during different growing seasons of Nigeria under RCP 2.6.

Projection of climate in Nigeria revealed significant changes in both rainfall and temperature for different RCPs. Annual rainfall was projected to change in the range of −7.5% to 27%. All the RCPs showed both increase and decrease. The temperature was likely to increase in the range of 0 to 5.5 °C by different RCPs. The least increase was estimated by RCP 2.6 (0–4 °C), followed by RCP 4.5 (0–5 °C) and RCP 8.5 (0–5.5 °C). The spatial patterns of rainfall, temperature and the SPEI trends during different growing seasons for the period 2010–2100 are presented in Fig. 6 for RCP 2.6. The same scale is used to show the changes for comparing changes during different seasons. The spatial pattern of rainfall trend was found to vary significantly for different crop growing seasons while the temperature was found to increase and the SPEI is shown to decrease over the entire country for the growing seasons. As all crops are grown mostly around summer, the spatial pattern in the trend of climate (e.g. rainfall and temperature) and SPEI was not found to vary markedly. However, the magnitude of the changes was found to vary significantly due to large variation in the time spans of different growing seasons.

The rainfall during rice (N) growing season is expected to increase significantly in the northwest and southwest while a decrease is expected mostly in the southeast. On the other hand, rainfall in corn (N) season was found to follow a similar pattern to that of rice (N). The number of points, showing increase or decrease in rainfall, during different seasons however vary. Increase at more grid points was observed during corn (N) while the least number of increases at grid points was observed during corn (S) season. Decrease in rainfall was found at more grid points during rice (N) growing season and less number of grid points during corn (S). An increase in rainfall was also noticed in the central-west during rice (S) growing season, in the central-west, south and central-north during corn (N) and central-west during corn (S).

Increase in temperature is expected to be highest in the northwest and lowest in the southeast during most of the cropping seasons. However, there is some discrepancy in the pattern. It is expected to increase at higher rate in the northwest during rice (N) and corn (N) growing seasons while least during corn (S) and rice (S) seasons in the same region. Decrease in the SPEI is expected to be highest in the north, particularly in the northwestern parts and least in the south–south, southeast, and parts of southwest for most of the growing seasons. The SPEI values are expected to be lower in the regions where temperature would increase more and higher in the regions where temperature is likely to increase less.

Similar patterns of rainfall, temperature and the SPEI changes were observed for other RCPs. However, increase in rainfall is expected at more grid points under RCPs 4.5 and 8.5 compared to RCP 2.6. Also, decrease would occur at less grid points for these RCPs than for the RCP 2.6. The number of grid points at which significant changes in rainfall, temperature and SPEI was projected for different RCPs for the earliest (2010–2060) and the last (2050–2100) periods are presented in Table 4. This shows an increase in rainfall at more grid points and decrease at less grid point for RCP8.5, particularly at the end of the century (2050–2100). For example, rainfall during Rice (N) growing period was projected to increase at 24 grids and decrease at 15 grids under RCP2.6 while it was projected to increase at 28 grids and decrease at 4 grids under RCP8.5. In contrast, changes in temperature and the SPEI were found consistent for all the RCPs. Significant increase in temperature and decrease in SPEI was observed at most of the grid points during all cropping seasons under all the RCPs.

**Table 4:** The number of grid points at which rainfall, temperature and the SPEI were found to change significantly during different growing seasons for earliest (2010–2060) and last (2050–2100) periods for the RCP scenarios.

Crop	RCP	Period	Rainfall		Temperature		SPEI	
			+	–	+	–	+	–
Rice (N)	2.6	2010–2060	24	15	308	−	−	288
		2050–2100	2	14	312	−	−	294
	4.5	2010–2060	2	13	323	−	−	321
		2050–2100	28	4	323	−	−	306
	8.5	2010–2060	0	60	323	−	−	323
		2050–2100	18	8	323	−	−	323
Rice (S)	2.6	2010–2060	1	0	308	−	−	303
		2050–2100	17	13	322	−	−	323
	4.5	2010–2060	2	12	323	−	−	321
		2050–2100	38	12	323	−	−	311
	8.5	2010–2060	4	27	323	−	−	323
		2050–2100	46	11	323	−	−	323
Corn (N)	2.6	2010–2060	30	14	309	−	−	293
		2050–2100	2	24	318	−	−	322
	4.5	2010–2060	6	6	323	−	−	320
		2050–2100	34	5	323	−	−	318
	8.5	2010–2060	2	19	323	−	−	323
		2050–2100	35	22	323	−	−	323
Corn (S)	2.6	2010–2060	5	2	298	−	−	302
		2050–2100	43	6	300	−	−	322
	4.5	2010–2060	2	4	323	−	−	323
		2050–2100	42	1	323	−	−	319
	8.5	2010–2060	5	16	323	−	−	323
		2050–2100	66	2	323	−	−	323

### Time-varying changes in climate and drought index

**Figure 7:**
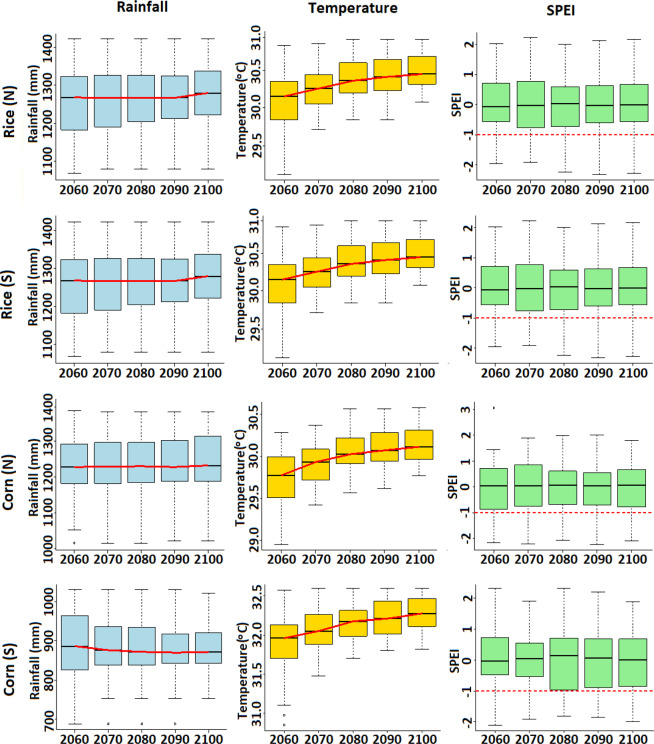
Changes in rainfall, temperature and the SPEI for different 50-year periods with a time step of 10-years for the period 2010–2100 during different growing seasons under RCP 2.6.

The changes in precipitation, temperature and SPEI for different 50-year periods with a time interval of 10-year over 2010–2100 during different growing seasons are presented in Fig. 7. It shows that there is no significantly change in rainfall during different growing seasons over the period 2010–2100. The rainfall is expected to be mostly constant during Corn (N) growing season, slightly increase in the last fifty years of the century for rice (N) and rice (S) and decrease in the early 50-year periods for corn (S). The height of the box was found to reduce gradually for most of the crops, except for corn (N). This indicates more homogeneity in the spatial distribution of rainfall in Nigeria during different crop growing seasons.

The temperatures are expected to increase over the entire century during different growing seasons. Increase is expected to be faster during early periods compared to the last part of the century. However, the rate of increase was found to be very similar for different growing seasons. It was found slightly higher for corn (N). The changes in rainfall and temperature would cause changes in the SPEI for different 50-year periods and growing seasons. The expected changes in the SPEI were also found to vary significantly for different crops. The mean SPEI for most of the crops was projected to increase during mid of the century and then decrease in the end, except for corn (N). For corn (N), the mean SPEI was found not to change over time. Though the mean SPEI was projected to change less, the height of the box showing the spatial variability of SPEI for different 50-year periods for different crops was projected to vary significantly. The spatial variability of SPEI is expected to decrease gradually for most of the crops except for corn (N). The reduction of spatial variability indicates more homogeneity in the spatial distribution of the SPEI with time in Nigeria. It means that the areas which were less prone to droughts would experience gradual increase in droughts in the future.

The time-varying change in rainfall, temperature and the SPEI for other RCPs were found to follow the similar pattern of RCP2.6. The subtle differences were sharper increase in temperature and a slight increase in rainfall in the later part of the century for higher RCPs. Consequently, the changes in the SPEI were not much different from RCP2.6 as the impact of sharp rise in temperature might be offset by increased rainfall.

### Influence of rainfall and temperature on the SPEI

**Figure 8:**
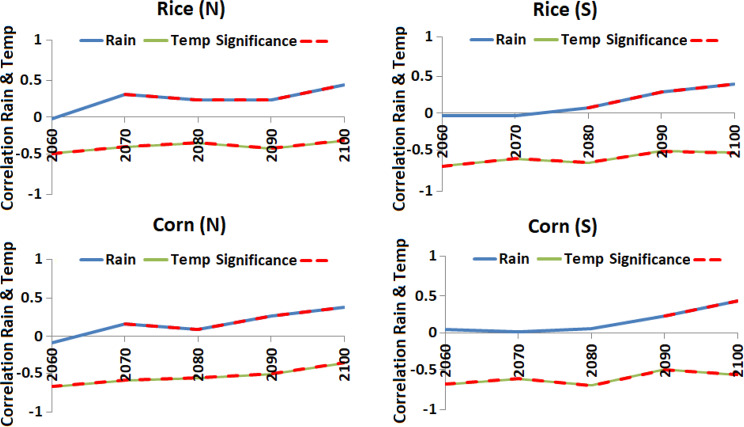
The correlation of the SPEI with rainfall and temperature for a 50-year moving window and a time step of 10-years over the period 2010–2100 during different crop growing seasons under RCP 2.6.

The correlation of the SPEI with rainfall and temperature for a 50-year moving window and a time step of 10-year over the period 2010–2100 during different crop growing seasons are shown in Fig. 8, which shows negative correlation of the SPEI with temperature. The correlation with temperature was also found significant for all the 50-year periods for all the crops. This indicates an increase in temperature would be a major cause of decrease in the SPEI in Nigeria in the coming years. The relation of the SPEI with rainfall was found significant only in the last parts of the century. The positive relationship indicates there would be a slight decrease in the SPEI at the end of the century. The results indicate temperature would play a vital role for increasing droughts under RCP2.6. Similar results were obtained for RCPs 4.5 and 8.5. However, drought severity was projected to decrease in the end of the century to the level of the early part of the century for RCP4.5 and 8.5 due to an increase in projected rainfall. Therefore, both rainfall and temperature would play a crucial role in defining drought severity at the end of the century for most of the cropping seasons for RCP4.5 and 8.5.

### Changes in drought return periods

**Figure 9:**
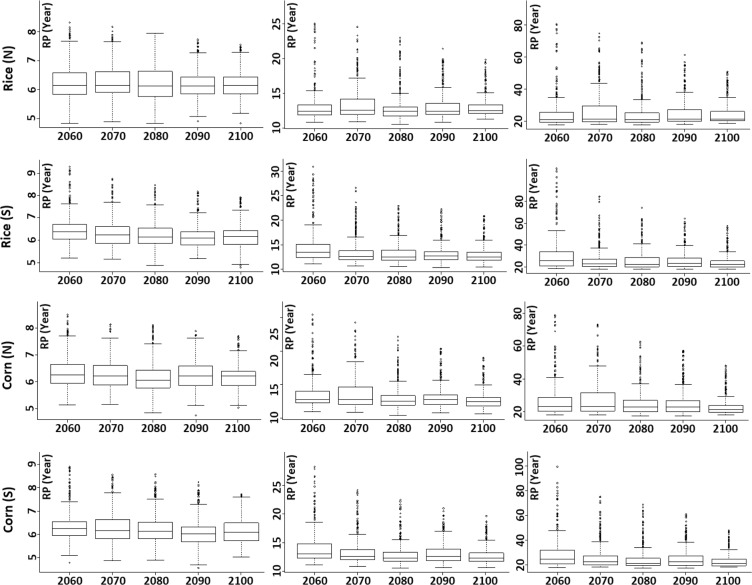
Return periods of moderate, severe and extreme droughts during different crop growing seasons for a 50-year moving window and a time interval of 10-year over the period 2010–2100 under RCP 2.6.

The return periods of different severities of droughts, i.e. moderate, severe, and extreme, estimated at different grids for different 50-year periods over the century (2010–2099) for different crops are presented for RCP 2.6 in Fig. 9. The longer size of the box or the longer extent of the whisker means that there is a wide range of droughts return periods while the shorter size or a shorter whisker displays there are closeness in the return periods of droughts.

Figure 9 further shows the median values of the return periods of rice (N) falling within the range of 6.10–6.30 for the moderate, 12.50–13.00 for the severe, and 21.5–23.5 years for extreme droughts. A close look at the median values shows that, overall, there is a decreasing trend towards the end of this century, meaning that the droughts are going to be more frequent. This is also observable from the narrowing of the whiskers over time. However, for rice (S) and corn (N), fluctuation in the median drought return period was observed which may be due to the variation in rainfall and temperature during different periods. The lower whisker of the box was not found to change for most of the crops while the upper whisker was found to shorten gradually. This indicates that the locations where drought return period is high or that are less prone to droughts would gradually become more prone to droughts due to a decrease in drought return period or an increase in drought frequency.

The changes in mean return periods of droughts (Table 5) revealed a small reduction in the return period of moderate and severe categories for all the cropping seasons compared to extreme droughts. For example, moderate droughts during rice (N) growing period was found to reduce from 6.25 years in 2010–2060 to 6.11 years in 2050–2099, severe droughts from 12.94 years to 12.24 years, while extreme droughts from 24.0 years to 21.01 years for RCP2.6. Similar pattern was observed for other crops, except for corn (N). The return periods of different categories of droughts during corn (N) was not found to change markedly. This indicates the impact of climate change on extreme droughts compared to other categories. Extreme droughts would be more recurrent for almost all the cropping seasons under RCP2.6.

**Table 5:** Median values of return periods for moderate, severe, and extreme droughts for different periods for all the cropping seasons.

Crop	Severity	RCP 2.6		RCP 4.5		RCP 8.5	
		2010–60	2050–2100	2010–60	2050–2100	2010–60	2050–2100
Rice (N)	Moderate	6.25	6.11	6.05	6.18	6.15	6.10
	Severe	12.94	12.24	12.41	12.59	12.30	11.75
	Extreme	24.00	21.01	21.11	22.14	21.12	19.98
Rice (S)	Moderate	6.21	6.19	6.16	6.13	6.13	6.06
	Severe	12.65	12.56	12.00	12.70	12.03	11.89
	Extreme	22.04	21.70	20.26	23.42	20.40	20.37
Corn (N)	Moderate	6.13	6.07	6.09	6.13	5.98	5.91
	Severe	12.65	12.40	12.19	12.94	12.07	11.61
	Extreme	21.97	21.74	20.39	24.24	21.19	19.98
Corn (S)	Moderate	6.27	6.22	6.13	6.13	6.28	6.17
	Severe	12.97	12.50	12.08	12.50	12.22	11.72
	Extreme	24.68	21.99	20.63	21.88	19.96	19.49

The changes in return periods for different categories of droughts for other RCPs are also shown in Table 5. The results were found similar to that obtained for RCP2.6. The impact of climate change on extreme droughts compared to other categories of droughts was also observed for RCP4.5 and 8.5. However, the impact was less for higher RCPs due to projection of more rainfall. Overall, the results revealed decrease in return period or increase in frequency of all categories of droughts for all the growing seasons under all scenarios. However, increase in drought frequency would be less for higher RCPs compared to baseline RCP of 2.6.

## Discussion

Water resources and agriculture constitute important aspects of sustainable existence of life, which are expected to be affected severely by climate change in many parts of the globe^13,77–80^. As there is a likelihood of increasing temperature and more erratic rainfall pattern across the world, assessment of regional climate and its consequence on drought could support effective management of water resources and agricultural practices.

Though many studies have been conducted on droughts in Nigeria, projection of the events is sparse, particularly during cropping seasons and using the CMIP5 GCMs. Some studies revealed that changes in rainfall and temperature could drive onset, frequency and severity of droughts across different settings^81–83^, which aligns with findings of this work. Abiodun *et al*.^40^ assessed the potential influences of global warming on future climate and extreme events in Nigeria using emission scenarios (B1 and A2) on the future climates (2046–2065 and 2081–2100). It was revealed that there would be an increase in temperature across all ecological zones of Nigeria, which may aggravate the frequency of extreme rainfall events in the south and southeast, and annual rainfall reduction in the northeast leading to floods and droughts, respectively. While this study aligns with some of their finding, it contradicts with decrease in rainfall in the southeast of the country. These differences may be due to the differences in the data and methods employed. However, both studies suggested increased droughts in the north compared to the south, indicating temperature will have substantial influence in defining droughts across Nigeria than rainfall. In the same region of West Africa, Oguntunde *et al*.^84^ projected the impacts of climate change on hydro-meteorological drought during 2046–2065 and 2081–2100 over the Volta Basin, using the SPI and SPEI, and found an increase in drought intensity and its spatial extent, which was highest for the SPEI. Compared to the present day episodes in the basin, drought frequency (events per decade) may be magnified by a factor of 1.2 (2046–2065) to 1.6 (2081–2100). This is corroborated by the decreasing trend in the box and whiskers plots (Fig. 9), and the decreasing median of drought return period particularly under RCP 2.6 and 8.5 (Table 5). A study in the Lower Mekong Basin (LMB)^85^, covering Thailand, Cambodia, Laos and Vietnam using the SPI method for the period 2016–2099, showed the region is expected to experience more severe and intense droughts; Ojeda *et al*.^86^ made use of the SPI and SPEI methods for future projection (2021–2050 and 2071–2100) of drought under RCPs 4.5 and RCP 8.5 over the Iberian Peninsula, and found that increase of drought event could be more evident using the SPEI, and there would be an increase in the frequency and severity of drought particularly under RCP 8.5 during the period 2071–2100. This study also suggested that droughts would be more severe under RCP 8.5 and the frequency of occurrence would be higher as indicated by the decreasing return period after the mid-century (Table 5).

Studies demonstrated that uncertainties in downscaling and climate modelling may stem from different products being used, including GCM initialization and parameterization^87^; future GHG emission and aerosols content scenarios^88^; approaches of climate downscaling^89^; and bias correction methods^90^. Prudhomme and Davies^91^, for instance, found that major contributor of uncertainty in climate change impact assessment is the GCMs. Jung *et al*.^92^ reported that the GCMs structure can increase uncertainty in evaluating climate impact. Sharma *et al*.^90^, on the other hand, speculated a significantly smaller contribution in uncertainty from a downscaled projection. This study, like the aforementioned ones, made use of the GCMs including 20 CMIP5 GCMs, as a result, the degree of uncertainties may have resulted from individual GCMs, suggesting that careful selection of the GCMs and bias correction method is important in projecting climate impacts.

Even though, CMIP5 GCMs are considered to be the most advanced models^93^, they are also subject to uncertainty^94^. Over the past two decades, several approaches are adopted to assess uncertainties in climate models. For example, a study by Aich, *et al*.^95^ employed a bias correction approach to evaluate uncertainties in projecting climate of large river basins of Africa. Their study confirmed the presence of large uncertainties despite removal of bias. Furthermore, other studies first removed bias of individual models and then developed multi-model ensemble to predict future scenarios, however large uncertainties associated with individual models were still reported Sa’adi, *et al*.^19^. In order to reduce uncertainties in future projection, their study suggested developing the MME by combining the projections of 20 CMIP5 GCMs using generalized linear model. Similarly, Pour, *et al*.^48^ followed a three- step approach to reducing uncertainties in projecting precipitation; the GCMs were first ranked based on their performance, support vector machine was then used to reduce bias in the top ranked four selected GCMs, and finally, an MME mean was generated from bias corrected GCMs using the RF. The GCMs were first ranked, in this present work, using entropy-based approaches to choose the GCMs that can best replicate observed rainfall and temperature properties. The bias correction approaches were subsequently applied to reduce bias in the selected GCMs. Finally, the RF was used to generate the MME to overcome bias in the selected GCMs. Due to uncertainties inherent in GCMs and other data used in this study (e.g., GPCC rainfall and CRU temperature) particularly during their preparations, uncertainties in the results cannot be ruled out. However, it can be remarked that careful selection of the GCMs and the use of approaches help reducing uncertainties to a certain extent as found here.

Despite the fact that mean yield level was the main focus of this work, interannual variability of agricultural production is highly influenced by climatic parameters. Due to data scarcity, the effects of droughts on interannual agricultural yield could not be considered. But existing works revealed that a decrease in seasonal surface water availability due to climate change seriously impacts rice cultivation in northern Italy^96^. Likewise, Zhang *et al*.^97^ reported that crop growth to water use efficiency (WUE) would breakdown under climate change scenarios. The resiliency of wheat to climate change showed a decline in wheat diversity in most European countries after 2002–2009^98–100^. Zhang and Huang^101^ assessed the impacts of climate change and inter-annual variability on cereal crops (maize, rice, and wheat) in China. Climate warming was observed to be significant, but there were no statistical significance trends in precipitation and solar radiation in most of China. Generally, maize was found to be particularly sensitive to warming. However, there was correlation of increase in temperature with both low and high yield of rice and wheat, which deviates from the current view that, warming results in decline in yields. Of the cereal crops, further analysis revealed that reduction in yields with higher temperature is accompanied by lower precipitation, which mainly occurred in northern parts of China, indicating droughts reduced yield due to lack of water resources. Agronomic measures are required for mitigation of impacts of climate change-induced droughts in crop yields. Kahiluoto *et al*.^100^ suggested a change in breeding programs and cultivar selection practices for adaptation to the uncertainty and variability in climate. Zampieri *et al*.^96^ highlighted the needs for better management of crops for mitigation of climate change impacts on crop yield.

Agriculture is one of the major sectors of Nigerian economy, contributing about 20% to gross domestic product (GDP), and more than 80% of rural population depends on this for their life and livelihoods. This sector, however, is significantly affected by climate change, and the impact could be severe in future than present anticipation. Many studies already reported the impact of climate change on agricultural productivity in the country^102–104^. Crop simulation models^105^ indicated that even an increased rainfall in many parts of Nigeria, there is no likelihood of offsetting expected crop yield reduction due to an increase in temperature, in particular over the medium-term period (until 2050), especially for cereals. In addition, there have been reports of contamination of agricultural soils with heavy metals which can affect crop yields^106,107^. These assert, in addition to other measures, the need for development of adaptation measures for combating climate change impacts and meeting growing demand of food.

## Conclusions

Meteorological droughts during growing seasons were projected for Nigeria using the SPEI and an ensemble of CMIP5 GCMs for three RCPs 2.6, 4.5 and 8.5. The results of this study revealed that temperature is the dominant variable that may lead to a decrease in the SPEI in Nigeria due to climate change. This is especially pronounced in the northwest part, where temperature may be high, which could lead to a decrease in SPEI under all RCPs. However, a combined effect of rainfall and temperature is likely to be prominent at some locations such as southeast where rainfall would be significantly decreasing during the growing seasons of rice (N) under RCP 2.6, and thus, highest decrease in the SPEI. This was also pronounced in southwest and south-south parts, where rainfall could increase significantly for the growing seasons of rice (N) and corn (N). Note that decrease in the SPEI values would not be high compared to other parts where temperature is the only determinant, affecting the SPEI. Generally, a decreasing trend in the SPEI for all the growing seasons was found to correlate strongly with the increasing trend of temperature, suggesting droughts are going to be more frequent in Nigeria under different RCPs. During the last 20 years of the century, an increasing trend in the return periods of droughts for most crops under RCP 2.6 was observed, meaning that droughts would be less frequent. This may be due to stabilization of temperature rise and expected increase in rainfall. Similar occurrences are projected for other cropping seasons under RCPs of 4.5 and 8.5. The northern semi-arid and arid regions, where rainfall is low and groundwater is declining fast, would be the most affected by droughts under all RCPs. The results, thus, reinforce observations of previous works, that variability of climate may enhance frequency and intensity of droughts in Nigeria. The methodology developed in this study can be used for reliable projections of drought characteristics in any region, and the results can be used in developing adaptation and mitigation plans in Nigeria.
